# Small Endoscopic Sphincterotomy plus Large-Balloon Dilation for Removal of Large Common Bile Duct Stones during ERCP

**DOI:** 10.12669/pjms.294.3662

**Published:** 2013

**Authors:** Qian Jun Bo, Xu Li Hua, Chen Tian Min, Gu Liu Gen, Yang Yan Mei, Lu Hua Sheng

**Affiliations:** 1 Qian Jun Bo, Department of Gastroenterology, The First People’s Hospital of Nantong, Nantong, China 226001.; 2 Xu Li Hua, Department of Gastroenterology, The First People’s Hospital of Nantong, Nantong, China 226001.; 3 Chen Tian Min, Department of Gastroenterology, The First People’s Hospital of Nantong, Nantong, China 226001.; 4 Gu Liu Gen, Department of Gastroenterology, The First People’s Hospital of Nantong, Nantong, China 226001.; 5 Yang Yan Mei, Department of Gastroenterology, The First People’s Hospital of Nantong, Nantong, China 226001.; 6 Lu Hua Sheng, Department of Gastroenterology, The First People’s Hospital of Nantong, Nantong, China 226001.

**Keywords:** Small endoscopic sphincterotomy plus large-balloon dilation, Endoscopic sphincterotomy, Common bile duct stones, Mechanical lithotripsy, Endoscopic retrograde cholangiopancreatography

## Abstract

***Objective:*** This study compared the therapeutic benefits and complication rates of small endoscopic sphincterotomy plus large-balloon dilation (ESLBD) with those of endoscopic sphincterotomy (EST) alone for large bile duct stones.

***Methods:*** We compared prospectively ESLBD group (n=63) with conventional EST group (n=69) for the treatment of large bile duct stones (≥15mm). Mechanical lithotripsy was performed when the stone could not be removed using a normal basket. We compared the rates of stone removal, frequency of mechanical lithotripsy use, procedure-related complications, and recurrent stones.

***Results:*** A total of 132 patients were reviewed in the study. The mean age of the patients was 67.9 years. The two groups showed significant differences in complete stone removal during the first session (80.9 vs. 60.8%; P = 0.046), the use of mechanical lithotripsy (7.94 vs. 24.6%; P = 0.041), and less duration of admission (P =0.045). After ERCP, there were some instances of oozing in both groups, All patients recovered completely, 14 patients had recurrent common bile duct stones among the follow-up duration.

***Conclusion:*** The ESLBD technique seems to be a feasible and safe alternative technique for conventional EST and EBD and has no more Post-ERCP complications.

## INTRODUCTION

Treatment of common bile duct stones (CBDS) is one of the main indications for Endoscopic retrograde cholangiopancreatography (ERCP). Approximately 5 to 10% of duct stones cannot be removed using either EST or EBD alone.^1^ Stone extraction failure generally occurs with stones larger than 1.5 cm. Small endoscopic sphincterotomy plus large-balloon (12–20 mm) dilation (ESLBD) has recently been introduced as an adjunctive technique to enlarge the papillary orifice after EBS in order to facilitate removal large or difficult stones. We conducted a prospective randomized study to compare the therapeutic benefits and complications of ESLBD with conventional EST for the treatment of large (≥ 15 mm) common bile duct stones.

## METHODOLOGY

***Patients: ***Inclusion criteria were as follows: age 18 years or older, ability to give informed consent. The exclusion criteria for this study were the following:^2^ (1) bleeding tendency with INR >1.5, (2) platelet count <50×10^9^/L, (3) anticoagulation therapy within 72 h of the procedure, (4) bilio-colic fistula, (5) stone size >50 mm, (6) acute cholecystitis, (7) acute pancreatitis, (8) cholangitis, (9) intrahepatic duct stones, (10) pancreatobiliary malignancy, and (11) surgical history involving the biliary tree (not including the gall bladder) or gastrointestinal tract, such as the stomach or small bowel, which can alter the papillary location.

From January 2008 to January 2012, 132 patients were enrolled into this prospective study at The First People’s Hospital (a teaching hospital in NanTong City, China). All patients were diagnosed as having common bile duct stones by endoscopic retrograde cholangiography (ERCP) or magnetic resonance imaging (MRI), and the maximum stone of every patient was at least 15 mm in maximum diameter.

There were 63 patients in ESLBD group and 69 patients who underwent conventional EST alone. The ages of the patients ranged between 25 and 86 years, and the mean age was 67.9± 23.3. Their demographic characteristics are presented in Table-I.

This study was approved by the ethics committee of our hospital, and all patients provided written informed consent before entering the study.

**Fig.1:**
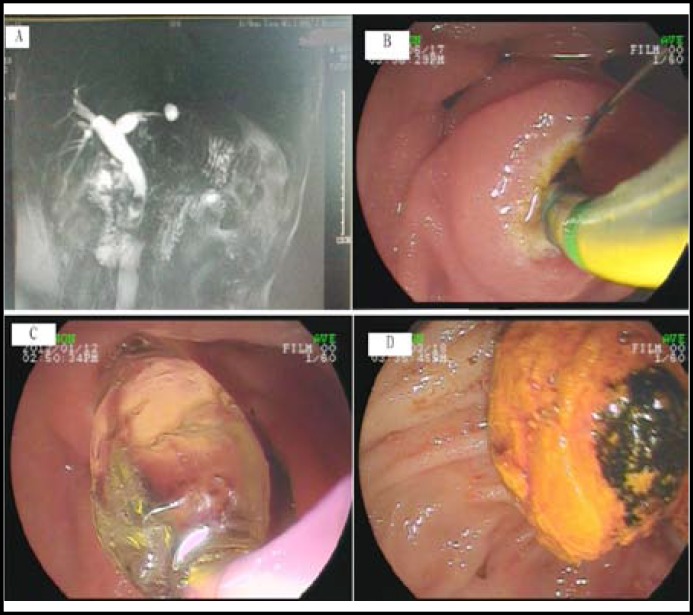
Small endoscopic sphincterotomy plus large-balloon dilation (ESLBD).

**A:** One free-floating large stone (14mm*16mm diameter) was confirmed by magnetic resonance imaging.** B: **A minor incision of up to one-third of the papilla was performed over a guidewire.

**C:** After small EST, EBD with a balloon catheter (max 20 mm in a diameter) was performed.

**D:** CBD stone was retrieved by basket through the dilated orifice of major papilla.

**Table-I:** Baseline Characteristics of the Patients undergoing endoscopic retrograde cholangiopancreatography (ERCP).

	*ESLBD (n= 63)*	*EST alone (n=69)*	*P value*
Age(years) ^☆^	67.3 ± 23.4	68.4 ± 22.8	0.233
Sex(M/F)	32/31	36/33	0.065
Mean diameter of CBD (mm) ^☆^	22.4 ± 7.3	21.5 ± 6.5	0.178
Mean diameter of CBD stones (mm) ^☆^	20.6 ± 5.4	20.3 ± 5.3	0.402
Mean number of stones (Single/Multiple) ^☆^	2.2±1.2(20/41)	2.3 ± 1.3(22/46)	0.739
CBD angle < 120°^☆^	12	14	0.428
Intact gall bladder (Gall bladder stones)	47 (28)	50 (32)	0.849
Juxtapapillary diverticulum	19	21	0.613
Distal CBD tapering	11	13	0.361
Symptoms			
Pain	45	48	0.188
Fever	23	27	0.169
Jaundice	38	41	0.065
Pancreatitis	8	11	0.889
Serum WBC counts (*10^9^/L) ^☆⊙^	11.1 ± 6.5	11.2 ± 5.8	0.980
Serum Platelet (*10^9^/L) ^☆◇^	211 ± 95	220 ± 107	0.255
Serum amylase level (u/dL) ^☆^^¤^	150.7±88.7	164.9±96.3	0.236
ALT(IU/L) ^☆^	190 ± 135	188 ± 168	0.249
AST(IU/L) ^☆^	164 ± 142	175 ± 137	0.310
Alk-P(IU/L) ^☆^	312 ± 226	299 ± 230	0.272
r-GT(IU/L) ^☆^	524 ± 405	519 ± 381	0.206
Total bilirubin(umol/L) ^☆^	61.2 ±56.4	60.6 ±54.6	0.224

☆: Mean ± standard deviation, ⊙:Normal serum white blood cell (WBC) range:4 * 10^9^ –10 *10^9^ /L, ◇:Normal serum Platelet range:100 * 10^9^ –300 *10^9^ /L,¤: Normal serum amylase range:40–180u/dL,Somogyi.

**Table-II:** Results of endoscopic stone removal after ESLBD vs EST alone (stone size ≥ 15 mm)

	*ESLBD(n = 63)*	*EST alone(n = 69)*	*P value*
Complete stone removal			
(First session)	51	42	0.046
(Second session)	9	21	
(Total)	60	63	0.839
Mean number of required endoscopic sessions (Successful case)	1. 14 ± 0.36	1.23 ± 0.45	0.669
Number of mechanical lithotripsy	5	17	0.043
Pancreatic duct visualization	7	14	0.377
Mean procedure time (min) ^☆^	14.5 ± 8.4	15.9 ± 8.8	0.225
The duration of admission(days)	10.5± 6.6	14.9 ± 7.8	0.045
Post-amylase level (6 h) (IU/L) ^☆^	245.7 ± 233.6	258.3 ± 247.1	0.673
Post-amylase level (24 h) (IU/ L) ^☆^	357.4± 294.5	378.8 ± 290.9	0.718
Post amylase level (48 h) (IU/ L) ^☆^	236.3± 117.7	265.2 ± 157.0	0.504
WBC counts (*10^9^) (24 h) ^☆^	12.5±6.6	12.8± 6.8	0.539
WBC counts（*10^9^) (4 8h) ^☆^	9.3 ± 4.5	10.0± 6.6	0.676
Mean duration of follow-up (months) ^☆^	28.4 ± 18.5	28.8 ± 19.2	0.453

ESLBD: Small endoscopic sphincterotomy plus large-balloon dilation, EST: endoscopic sphincterotomy, ☆ Mean ± standard deviation.

**Table-III:** Complications in ESLBD and EST alone

	*ESLBD(n= 63)*	*EST alone(n= 69)*	*P value*
Perforation	0	1	
Cholangitis	1	1	0.942
Pancreatitis	4	6	0.387
Mild	3	4	
Moderate	1	2	
Severe	0	0	
Significant bleeding（need blood transfusion）	0	0	
Procedure-related mortality	0	0	
Total complication	5	8	0.790
Procedure-related oozing	14	21	0.642
Asymptomatic hyperamylasemia	9	16	0.482
Mean duration to recurrence CBDS (months) ^☆^	26.2 ± 16.7	25.3 ± 15.8	0.235
Patients of recurrent CBDS	6	8	0.436
cholangitis during follow-up	4	6	0.318
bile duct stenosis during follow-up	0	0	
acute cholecystitis during follow-up	0	0	

CBD: common bile duct, ☆: Mean ± standard deviation

*M* ***aterial: ***Management such as pharyngeal anesthesia and premedication before the procedure was carried out in the same manner as for general endoscopy, and all ERCPs were performed with a side-viewing endoscope (TJF-240, Olympus Optical Co, Ltd, Tokyo, Japan). The electrosurgical unit (ERBE VIO300, ERBE, Tubingen, Germany) for EST was used at a setting of Endocut I, effect 3 (output limit, 120 W). The patients were sedated with a standard dose of midazolam and meperidine.

***Study Design: ***Two groups were formed: the EST group and the ESLBD group, using opaque sealed envelopes according to a computer-generated randomized set of numbers. There were 69 patients who underwent only conventional EST in the EST group, and 63 patients underwent ESLBD in the ESLBD group. Stone size and number and bile duct size were documented on the cholangiogram during ERCP. Stone/bile duct size was assessed by comparing the largest diameter of the stone/bile duct with the diameter of the TJF240 endoscope, as measured on the cholangiogram.

In the EST group, the EST procedure was performed according to the conventional method with a pull-type sphinctertome, accomplished by extending the incision up to the major horizontal fold crossing the intramural portion of the bile duct (major EST).^1^

In the ESLBD group, the length of the sphincterotomy incision was limited to one-third that in the minor EST group. The minor EST was made from the orifice of the papilla proximally but did not extend beyond the horizontal fold or the transverse fold of the papilla. Then we inserted a CRE balloon (12–20 mm, Boston Scientific, Natick, MA, USA) over a guidewire. The balloon was inflated gradually with diluted contrast medium under fluoroscopic guidance to observe the gradual disappearance of the waist in the balloon and mucosal tearing of the duodenal papilla. Once the waist disappeared, the balloon remained inflated for 30s^1^ (Fig.1).

After the endoscopic procedure, the patients were kept in the hospital for at least two days under observation to determine whether pancreatitis or other complications arose. All patients received prophylactic antibiotics immediately after the procedure.

***Complications: ***Post-ERCP pancreatitis^3^ was defined as persistent abdominal pain of more than 24 hour, associated with serum amylase concentration more than three times the upper limit of normal. Hyperamylasemia was defined as any increase in the amylase levels above the normal limit without other symptoms.^4^^,^^5^ Hemorrhage was recorded only if there was clinical (not just endoscopic) evidence of bleeding, such as melena or hematemesis, with an associated decrease of at least 20g/L in the hemoglobin concentration or the need for a blood transfusion.^4^^,^^5^ Cholangitis was defined as a fever in the temperature above 38°C accompanied by leukocytosis and right upper quadrant pain after the procedure, it was thought to have a biliary cause without concomitant evidence of acute cholecystitis, All complications were classified and graded according to the consensus guidelines with some modification.^5^

***Statistical analysis: ***The continuous variables are expressed as means ± standard deviations or as medians with ranges. Statistical analysis was performed using the Chi-square test for non-continuous variables and Student’s t-test for continuous variables. The analyses were performed using SPSS 12.0 (SPSS Inc, Chicago, IL, USA). P < 0.05 was considered statistically significant.

## RESULTS

A total of 132 patients were enrolled in the current study. The patients’ demographic characteristics are summarized in Table-I, Results of endoscopic stone removal after ESLBD vs EST alone (stone size ≥ 15 mm) are shown in Table-II, Complications in ESLBD and EST alone are compared in Table-III. 

The ESLBD group consisted of 32 men and 31 women, and the EST group consisted of 36 men and 33 women. The two groups did not differ statistically in demographic characteristics, such as terms of Symptoms, Serum amylase level, presence of Juxtapapillary diverticulum, size of stones, number of stones, or CBD angulation less than 120°and so on. 

In the first session of endoscopic treatment, the success rate of complete retrieval of the CBDS was significantly higher in the ESLBD group (80.9%, 51/63) than in the EST-only group (60.8% 42/69) (P=0.046). The overall stone clearance was ultimately similar between the ESLBD group (95.2%, 60/63) and the EST group (91.3%, 63/69) (P=0.839) (Table-II). The number of required endoscopic sessions for complete stone removal were fewer in the ESLBD group (1.14 ± 0.36) than in the EST group (1.23 ± 0.45) (P=0.669), but not significantly difference. The use of mechanical lithotripsy tended to be fewer in the ESLBD group (7.94%, 5/63) than in the EST group (24.6%, 17/69) (P=0.041). The duration of admission was significantly shorter in the ESLBD group (10.5± 6.6 days) than in the EST group (14.9 ± 7.8 days) (P=0.045). 

Mean duration of the whole procedure was similar between the ESLBD group (14.5 ± 8.4 minutes) and the EST group (15.9 ± 8.8 minutes) (P = 0.225).

If incomplete stone clearance was suspected at the cholangiogram during ERCP, an endoscopic nasobiliary drainage catheter or a 10-Fr plastic stent was placed. Among those patients not successful (9/132), the failure was due to either stone impaction (6) or intolerance of patients (3), with two needed placement of a plastic stent, five were placed endoscopic nasobiliary drainage (2 in the ESLBD group and 3 in the EST group) and two were sent for surgical treatment (2 all in the EST group).

There were no statistical difference between the two groups in Post-amylase level (6 h, 24h, 48h), WBC counts (*10^9^) (24 h, 48h) (Table-II) , rate of recurrent CBDS and mean duration to recurrent CBDS (Table-III). The EST and ESLBD groups did not differ significantly in terms of total complication rates (7.9 % vs. 11.6%; P=0.790) (Table-III). As regards ERCP-related pancreatitis, hemorrhage, perforation, and cholangitis, the two groups were similar. All cases of pancreatitis were mild or moderate and self-limiting.

Perforation was observed in only one EST patient, who underwent an operation and then recovered. All patients recovered completely after conservative and endoscopic treatment respectively, and no procedure-related mortality was noted. Every patient had a mean follow-up duration of 28 months or more, of whom, 14 patients had recurrent CBDS (ESLBD: 6, EST:8). The mean duration of recurrence of CBDS was similar between the two groups.

In the study, one patient in the ESLBD group and three patients in the EST group developed Moderate-grade post-ERCP pancreatitis. Minor oozing that spontaneously stopped during the procedure was noted. Other fatal complications such as significant bleeding (need blood transfusion) or severe pancreatitis did not occur. However, asymptomatic elevation of serum amylase was noted in 10.6% (14/132) of the patients. The elevated serum amylase usually normalized within three days after the procedure and did not affect the clinical course of the patients. 

## DISCUSSION

EST is the most frequently used and the best nonsurgical treatment for the clearance of stones from the bile duct. Its success rate exceeds 90%.^2^^,^^6^^-^^10^ However, EST is still associated with an 8%-12% complication rates.^11^ Conventional EBD was introduced as an alternative method for the retrieval of CBDS, it did not involve cutting the biliary sphincter, and preserving its function. However, major limitations of EBD is difficulty in removing large stones (≥10 mm in diameter) and a high incidence of pancreatitis (5% to 15%).^1^^,^^12^^,^^13^ The risk of pancreatitis with EBD seems to be related to the pressure loaded on the orifice of the main pancreatic duct during balloon dilation.^14^

In order to shorten the procedure time as well as minimizing the chance of complications, simplifying the procedure while maintaining the effectiveness of large bile duct stones removal is warranted. ESLBD was first performed by Ersoz et al in 2003^15^, They got an 83% success rate in the first session. 

In our study, the overall rate of complete stone clearance was similar in the two groups (95.2 vs. 91.3%, respectively; P=0.839). The initial success rate for the removal of CBDS was significantly higher in the ESLBD group (80.9%) than in the EST-alone group (60.8%) (P=0.046), These data are similar to previously published results^1^^,^^14^^,^^16^^,^^17^, but contrary to what was reported by Kim HG.^2^ The mean number of required endoscopic sessions for complete stone clearance were less frequent in the ESLBD group (1.14±0.36) than in the EST group (1.23±0.45), but the difference was not statistically significant (P=0.669).

The use of mechanical lithotripsy in ESLBD (7.94%) was lower than in EST alone (24.6%) (P=0.041), the result was similar to previously published result.^1^ Therefore, it was thought that large-balloon dilation can dilate the orifice of the papilla more than conventional EST. But recent studies have reported that the need for mechanical lithotripsy is similar and there is no difference between the two groups.^6^^,^^8^ Kim HG^2^ reported that the outcome for large stone removal by endoscopic large-balloon dilation (ELBD) following EST remained controversial.

## CONCLUSION

In conclusion, we found that the overall stone clearance and complication rates were similar, but that the success rate of first session for complete stone removal and the frequency of mechanical lithotripsy might be statistically different for ESLBD than for EST alone. ESLBD would theoretically combine advantages of sphincterotomy and balloon dilation by increasing the efficacy at stone extraction while minimizing complications of either EST or EBD. ESLBD may be an effective, simple, safe treatment for removing large CBDS, especially for the removal of large (≥ 15 mm) bile duct stones. The procedure is technically easy, especially for the unskilled endoscopist, and it may reduce the need for mechanical lithotripsy. However, more larger studies are required to clarify the difference in the efficacy of the two procedures.

## Author contributions

**Qian Jun Bo**: Design of the study and endoscopy procedures. 

**Xu Li Hua**: Theoretical guidance, design of the study.

**Chen Tian Min**: Endoscopy procedures, collection of data getting informed consent from the patients, and approval by the Hospitals’ Ethics Committee.

**Gu Liu Gen**: Statistical analysis and interpretation of the data..

**Yang Yan Mei**: Following up the data of the patients.

**Lu Hua Sheng**: Endoscopy procedures, theoretical guidance.
